# Surgical treatment of scoliosis in a rare disease: arthrogryposis

**DOI:** 10.1186/1748-7161-5-24

**Published:** 2010-11-09

**Authors:** Tiziana Greggi, Konstantinos Martikos, Emanuela Pipitone, Francesco Lolli, Francesco Vommaro, Elena Maredi, Stefano Cervellati, Mario Di Silvestre

**Affiliations:** 1 Spine Surgery Division, Rizzoli Orthopaedic Institute, Bologna, Italy; 2 Scoliosis Center, Hesperia Hospital, Modena, Italy

## Abstract

**Background:**

The reported incidence of scoliosis in arthrogryposis varies from 30% to 67% and, in most cases, the curves progress rapidly and become stiff from early age.

The authors report six cases of scoliosis in arthrogryposis to assess the role of surgical treatment.

**Methods:**

Six cases (3 males, 3 females; mean age at surgery 13.2 years) with arthrogryposis multiplex congenita associated with the characteristic amyoplasia were reviewed: they were operated on for scoliosis at the authors' Spine Surgery Department between 1987 and 2008.

Surgery was performed using the Harrington-Luque instrumentation (2 cases), the Luque system (1), a hybrid segmental technique with hooks and screws (1) and spinal anchoring with pedicle screws (2).

**Results:**

The patients were clinically and radiologically reviewed at a mean follow-up of 4.2 years, ± 2.7 (range, 1 to 9 years). Three minor postoperative complications were encountered; a long-term pulmonary complication was seen in one case after reintervention and was successfully resolved after 10 days. Surgery was successful in the other 5 cases, where solid arthrodesis was achieved and no significant curve progression was observed at follow-up.

**Conclusions:**

The experience acquired with the present case series leads the authors to assert that prompt action should be taken when treating such aggressive forms of scoliosis. In case of mild spinal deformities in arthrogryposis, brace treatment should be attempted, the evolution of the curves being unpredictable; however, when the curve exceeds 40° and presents with marked hyperkyphosis, hyperlordosis or pelvic obliquity, surgery should not be delayed.

## Introduction

The term "arthrogryposis" was first used by Stern early in 1923 [1] to define the characteristic clinical appearance of three children affected with multiple congenital contractures (MCC) that produced symmetrical motion limitation of proximal and distal joints. The syndrome was first described by Otto in 1841, under the term "congenital myodystrophy". In 1982 Hall et al introduced the term "amyoplasia", which describes the most common type of arthrogryposis, characterized by quadrimelic involvement and replacement of skeletal muscle by dense fibrous tissue and fat [2]. At present at least nine different types of amyoplasia are recognized. Nowadays the term *arthrogryposis *is commonly used as a generic expression to describe the typical clinical appearance of children with congenital non progressive MCCs; however, it does not define a specific pathological entity, since it represents the phenotypical manifestation of more than 150 distinct entities [3], such as myelomeningocele, Larsen syndrome, multiple pterygium syndrome (Escobar syndrome), Freeman-Sheldon syndrome (whistling face syndrome), Beals contractural arachnodactyly, sacral agenesis, diastrophic dysplasia, metatropic dysplasia, thrombocytopenia-absent radius (TAR) syndrome, Steinert myotonic dystrophy, spinal muscular atrophy, congenital muscular dystrophy and Moebius syndrome. Various different efforts have been made in order to examine the various different etiopathological causes underlying the development of congenital MCCs and several subgroups have now been identified on a genetic basis [2,4,5]; the terminology defining the concept of arthrogryposis, however, is constantly being revised because of the constantly increasing knowledge on its developmental features.

Arthrogryposis occurs in approximately 1 in every 3000 newborns, caused by sporadic gene mutation or following a Mendelian pattern of inheritance (autosomal dominant, autosomal recessive or X - linked) [6,7]. Clinical manifestations of arthrogryposis include deforming joint contractures; in most cases (84%) both upper and lower limbs are involved, although only upper (5%) or lower (11%) limbs can be affected [8].

The reported incidence of scoliosis varies from 30% to 67%. Only a few studies in the literature have described spine deformities, as well as treatment options and results, in patients with arthrogryposis multiplex congenita [9-16]. In most cases, the curves tend to progress rapidly and become stiff from early age.

The present paper reports the results obtained in the surgical treatment of scoliosis in 6 children with arthrogryposis multiplex congenita, who have been treated at the authors' Division over the last 3 decades.

## Materials and methods

Six patients (3 males, 3 females) with arthrogryposis, who underwent major surgery for scoliotic curves at the authors' Division between December 1987 and May 2008 were reviewed. Mean age at first surgery was 13.1 years (range, 8 to 18 yrs); mean BMI was 19.9 (range, 15 to 29). Patients were clinically and radiologically reviewed at a mean follow-up of 4 years and 10 months (range, 1 to 11 yrs). In all cases the deformity progressed rapidly and by early adolescence it was already severe and stiff. The curve patterns were the following: a single large thoracolumbar curve in 4 cases (3 right convex, 1 left convex) and, in the remaining 2, a large thoracolumbar structured primary curve associated with a mild non-structured secondary (compensatory) curve (1 proximal thoracic and 1 distal lumbar). The preoperative mean Cobb angles of primary and compensatory curves was 85° (range, 42° to 111°) and less than 30° (17° in one case, 28° in the other), respectively. Hyperkyphosis was observed in 2 cases (74° and 73°); lumbar lordosis exceeded normal values in 5 cases; severe hyperlordosis was seen in 2 patients (103° and 82°) (Table 1). At the time of surgery, mean skeletal maturity according to Risser sign was 2.8 (range, 0 to 5): this finding, associated with the above mentioned radiographic data, confirms the rapid progression of the deformity right from early age.

**Table 1:** Preoperative values; radiographic postoperative and follow-up values in the same column

Patient	PREOP primary curve	PREOP secondary curve (where present)	PREOP lumbar lordosis	PREOP thoracic kyphosis	PREOP pelvic obliquity	POSTOP primary curve - FU primary curve	POSTOP secondary curve - FU secondary curve	POSTOP lumbar lordosis - FU lumbar lordosis	POSTOP thoracic kyphosis - FU thoracic kyphosis	POSTOP pelvic obliquity - FU pelvic obliquity
**Case 1 **9 yr FU	70°	-	103°	49°	9°	23° - 30°	-	59° - 76°	20° - 36°	3° - 3°

**Case 2 **6 yr FU	111°	-	56°	49°	28°	84° - 89°	-	37° - 38°	19° - 19°	19° - 22°

**Case 3 **3 yr FU	42°	17°	31°	21°	31°	26° - 31°	13° - 19°	31° - 31°	29° - 25°	11° - 19°

**Case 4 **4 yr FU	88°	28°	46°	50°	0°	79° - 96°	25° - 35°	34° - 40°	38° - 41°	6° - 6°

**Case 5 **2 yr FU	100°	-	82°	73°	18°	65°-69°	-	61°-63°	39°-45°	8° - 6°

**Case 6 **1 yr FU	99°	-	55°	74°	11°	82° - 85°	-	52° - 54°	60° - 60°	7° - 6°

A mild intellectual deficit was found only in 1 case, while the remaining children performed age-related intellectual and social activities, and attended classes as per their chronological age.

Lower limb deformities were observed in all of the patients who underwent at least one operating procedure for bilateral clubfoot in early pediatric age. Bilateral elbow tenolysis was performed in 2 subjects, bilateral knee arthromyolysis for non - traumatic subdislocation in 1 and surgery for bilateral hip non - traumatic subdislocation and palatoschisis in 1. All of the children except one could walk indoors and outdoors. One child had received Growth Hormone treatment since pediatric age. Spirometric evaluation revealed a severely restrictive pulmonary deficit in 3 cases and normal values in one: the first two patients had been treated in the early 80s and their lung capacity had not been instrumentally assessed; however, the clinical records from the authors' medical archive revealed decreased respiratory capacity.

After surgery, all of the patients were admitted to the ICU for careful monitoring of their conditions and pain therapy under anesthesiology.

Full-time brace was applied for at least 2 years before surgery in all of the cases, though with little impact on curve progression.

### Presentation of cases

**Figure 1:**
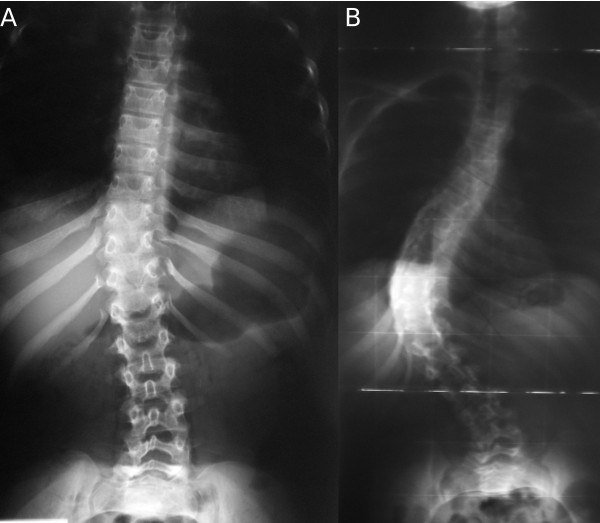
**(A, B), Case 1: Spine radiographs at the age of 1 year and 14 years, respectively**.

**Figure 2:**
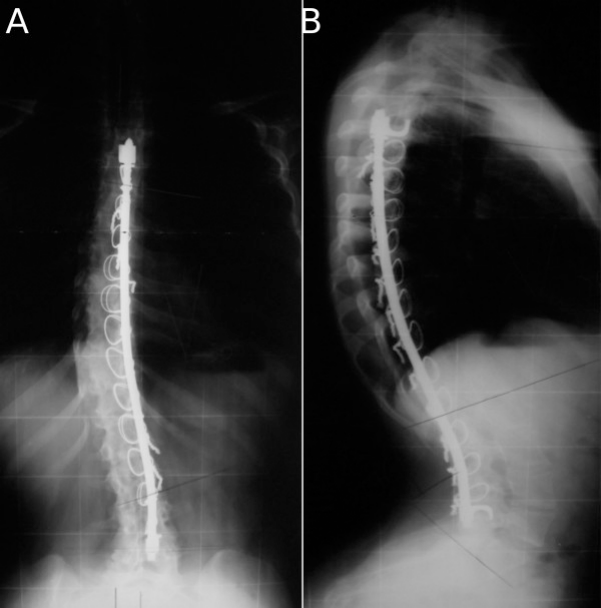
**(A, B), Case 1: Spine radiographs at a 9-year follow-up; anteroposterior (A) and laterolateral (B) view**.

**Case 1 **(female, born in 1973). The first patient treated at the authors' Division had multiple distal congenital contractures with a progressive, right convex, thoracolumbar scoliosis. She had already undergone 4 operations for bilateral clubfoot deformity before the age of 4; she could walk only in domestic environment and the clinical history showed compromised respiratory capacity. At the age of 1, the radiographic evaluation of the spine had shown a 20° right convex scoliotic curve with mild lumbar hyperlordosis and pelvic obliquity of 5°; no congenital vertebral anomalies had been seen. By the age of 14 and with a Risser index of 4, despite the full-time brace, scoliosis had evolved rapidly into a structured and stiff deformity with a Cobb angle of 70° and multiple vertebral fusions near the curve's apex: she presented severe lumbar hyperlordosis of 103°, thoracic kyphosis of 49° and pelvic obliquity had increased to 9° (Figure 1A and 1B). In 1987, at the age of 14, a spinal arthrodesis was performed from T4 to L4 according to the Harrington-Luque technique. Postoperatively, the Cobb angle was 27°, lumbar lordosis 59°, pelvic obliquity 3° and thoracic kyphosis 20°. During surgery no complications were encountered; blood transfusion was performed in the immediate perioperative period. A cast orthosis was applied for the subsequent 6 months followed by a full-time treatment with Lyonnaise brace for 1 year. The patient was clinically reviewed for 9 years after surgery. At follow-up, no long-term complications were observed, correction was stable and, at the age of 23, the above mentioned values were practically unchanged (Figure 2A and 2B). Although the walking capacity had remained the same, the patient referred a significant improvement in the physical and emotional state, with a satisfactory quality of life both indoors and outdoors.

**Figure 3:**
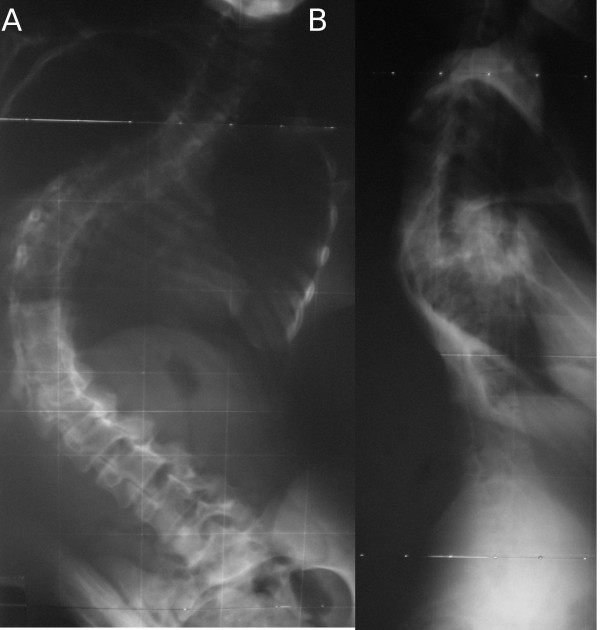
**(A, B), Case 2: Spine radiographs at the age of 15; anteroposterior (A) and laterolateral (B) view**.

**Figure 4:**
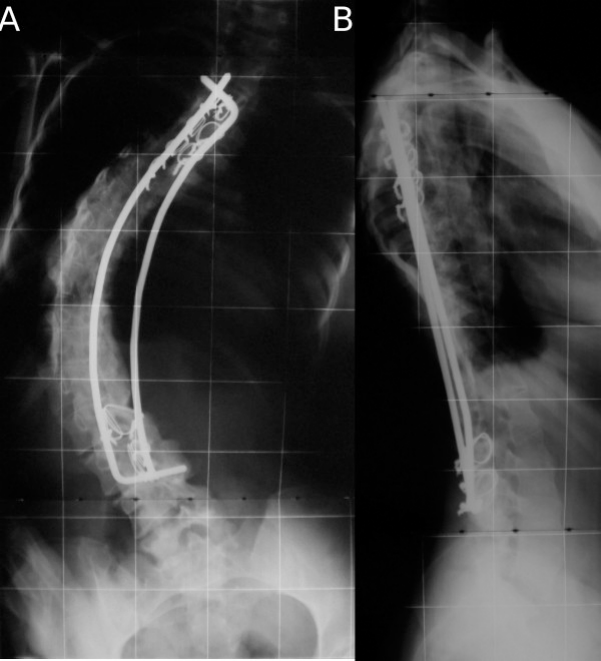
**(A, B), Case 2: Spine radiographs at a 6-year follow-up; anteroposterior (A) and laterolateral (B) view**.

**Case 2 **(female, born in 1976, patient with congenital arthrogryposis). Three months after her birth, x-ray examination of the spine had shown a very mild right convex thoracolumbar scoliotic deviation, which had developed into a curve of 27° with pelvic obliquity of 9° by the age of 6. Full-time brace treatment since the age of 6 had failed to control the progression of the deformity, since the following radiographic values were observed at the age of 15: a stiff structured 111° thoracolumbar scoliosis, 56° lumbar lordosis, 47° thoracic kyphosis, pelvic obliquity of 28° and a grade 3 skeletal maturity according to Risser (Figure 3A and 3B). By the age of 9, she had already undergone surgery twice for bilateral clubfoot congenital deformity and once for bilateral elbow tenolysis. Preoperatively BMI was 15, a severe respiratory deficit was observed and the patient could walk to a limited extent outdoors. Spinal arthrodesis with Luque instrumentation from T2 to L4 was performed at the age of 15, in 1991. Postoperatively, the Cobb angle was 84°, lumbar lordosis 37°, thoracic kyphosis 19° and pelvic obliquity decreased to 19°. A cast orthosis was then applied for 6 months, followed by a CLB brace for 2 years. At a 6-year follow-up, correction was stable and no complications were seen (Figure 4A and 4B).

**Case 3 **(female, born 1980). She had undergone her first clinical and radiographic evaluation at the authors' division at the age of 12, presenting a structured large right convex thoracolumbar scoliosis of 42° with a secondary non-structured left convex thoracic curve of 17, lumbar lordosis of 31°, a thoracic kyphosis of 21° and a significant pelvic obliquity of 31°. Until then, she had never been treated by orthopedic brace. By the age of 4, she had already undergone surgery 4 times for bilateral clubfoot deformity and could not walk. Preoperatively, BMI was 16.5 and the Risser sign 3. In 1992, at the age of 12 and after wearing a Risser cast for 30 days, she underwent posterior spinal T3-L5 arthrodesis with Galveston-Luque instrumentation. During the first 3 days after surgery, an urticarioid reaction of unknown cause was observed, associated with hyperpiressia, periorbital edema and tachycardia, which resolved after specific pharmacological treatment. Before hospital discharge, a cast orthosis was applied for 5 months, followed by a 2-year period with a bivalve brace. Postoperative radiographs showed the primary thoracolumbar curve had decreased to 26° and pelvic obliquity to 11°, whereas both lumbar lordosis and thoracic kyphosis had remained within physiological limits (31° and 29°, respectively). The secondary thoracic curve was equal to 13°. After a 3-year period, at the age of 15, the above mentioned values were still unchanged, except for the pelvic obliquity, which had increased to 19°. No long-term complications were observed, as well as no changes in the walking capacity throughout the years.

**Figure 5:**
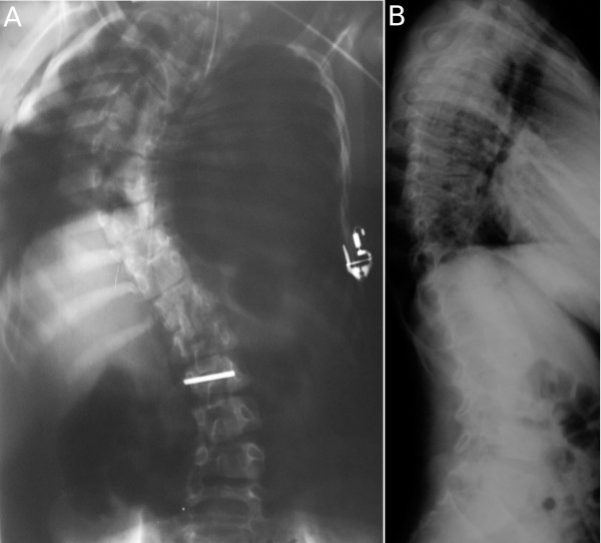
**(A, B), Case 4: Spine radiographs at the age of 8, anteroposterior (A) and laterolateral (B) view**.

**Figure 6:**
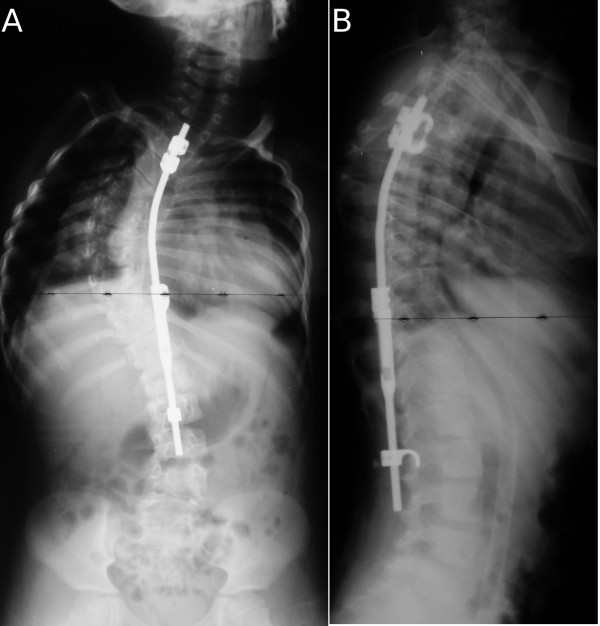
**(A, B), Case 4: Postoperative radiographs, anteroposterior (A) and laterolateral (B) view**.

**Figure 7:**
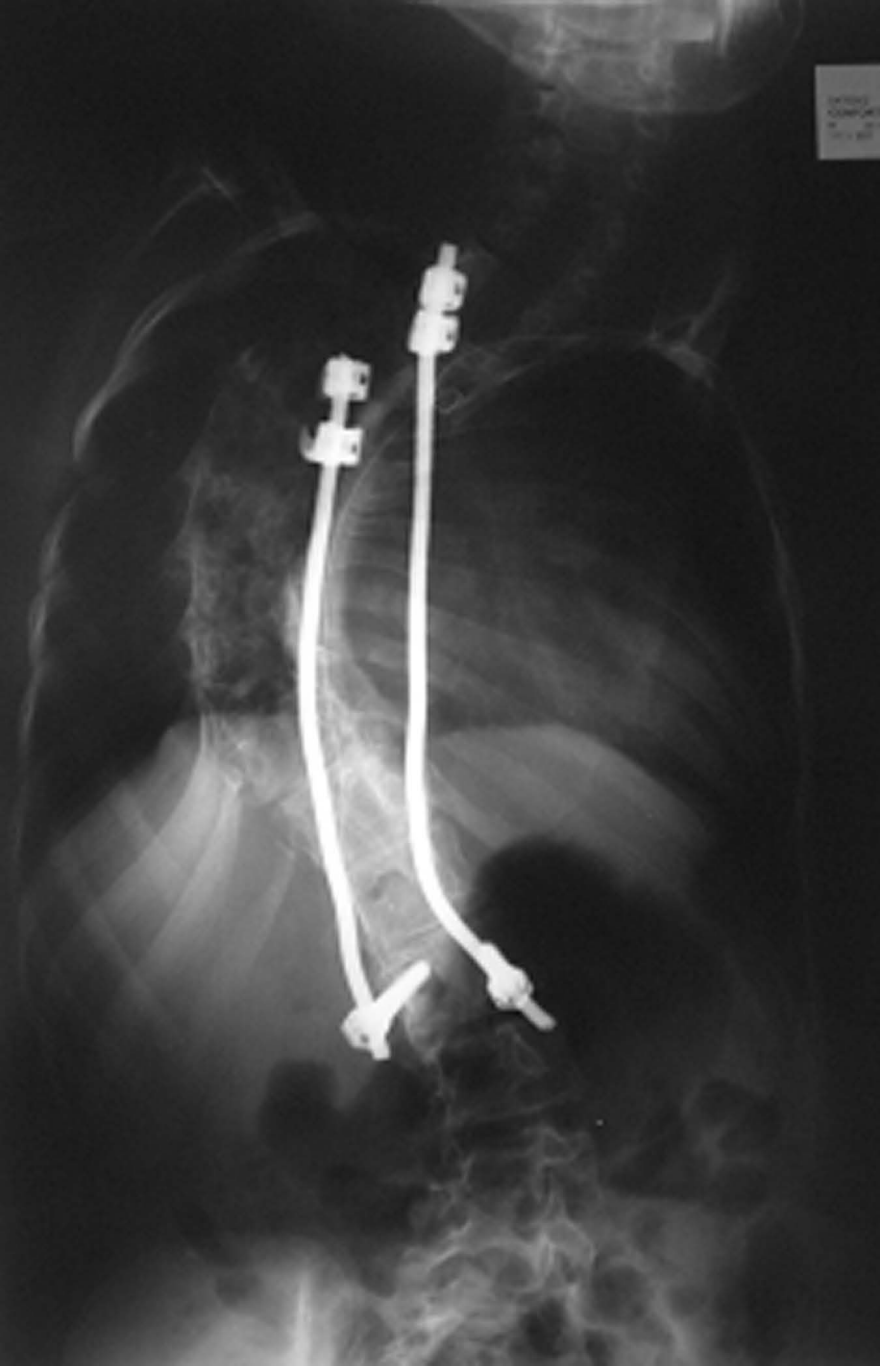
**Case 4: Spine radiographs at a 4-year follow-up after inserting the new Dual Rod instrumentation; anteroposterior view**.

**Case 4 **(male, born in 1990, patient with arthrogryposis). In pediatric age, he had received surgical treatment twice for bilateral clubfoot deformity and once for bilateral elbow tenolysis. The radiographic picture during pediatric growth had shown a rapid evolution of spinal deformity. He was admitted to the authors' division at the age of 8, when he presented with a large right convex thoracic curve of 88°, a left convex lumbar curve of 28°, lumbar lordosis of 46°, thoracic kyphosis of 50° and a well balanced pelvic obliquity (Figure 5A and 5B). Spirometric evaluation showed a severely restrictive respiratory deficit (FVC 32% and FEV1 35%). The patient had received no brace treatment before and a cast orthosis was applied for 7 months before surgery. At the age of 8, on account of the young age and the juvenile skeletal growth (Risser sign still 0), a combined single-stage surgery was performed, first by anterior approach for release from T4 to T8 and then by posterior approach from T3 to L2, instrumenting the patient with a single growing rod. No short-term complications were observed. Postoperative radiographic values did not significantly differ from the preoperative ones (thoracolumbar curve 79°, lumbar curve 25°, lumbar lordosis 31°, thoracic kyphosis 31°) (Figure 6A and 6B). After surgery, a cast orthosis was applied for 5 months, followed by a bivalve brace for the next 2 years. No complications were registered during this period. After two years, in 2000, at the age of 10, the patient underwent surgery for distal lengthening of the instrumentation and replacement of a dislodged distal hook. The radiographic picture at that time showed progression of the deformity (thoracic curve 96°, lumbar curve 25°, pelvic obliquity 11°). In 2002, at the age of 12, following hook dislodgment, the patient underwent a third surgery to remove the single bar and have it replaced with the dual rod system with proximal hooks and distal L2 screws; arthrodesis was performed with fresh-frozen banked bone. The deformity values were partially corrected as follows: thoracic curve 79°, lumbar curve 25° and pelvic obliquity 6° (Figure 7). In the immediate postoperative, pulmonary complications occurred with lung atelectasis, cough with haemoptysis and severe dyspnea: prompt treatment in ICU led to complete resolution of symptoms in 10 days. The patient considered his quality of life to be good after a 4-year clinical and radiographic follow-up period, and he could maintain his walking capacity in community.

**Figure 8:**
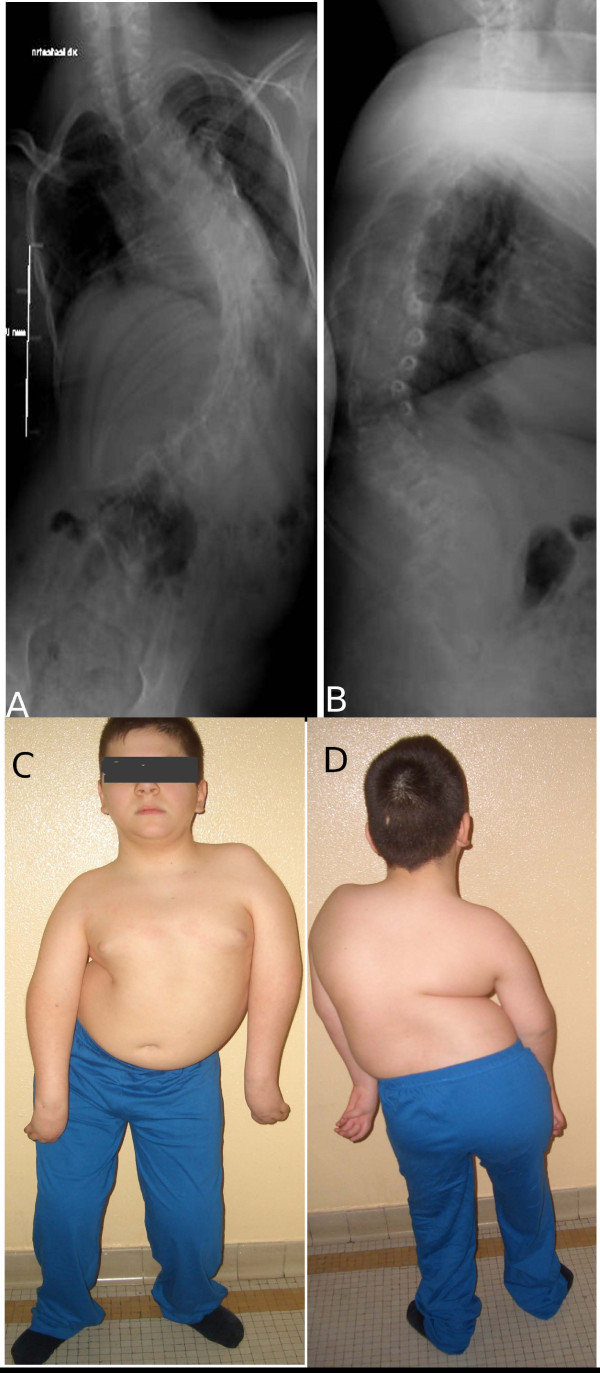
**(A, B, C, D), Case 5: preoperative, radiographic and clinical pictures**.

**Figure 9:**
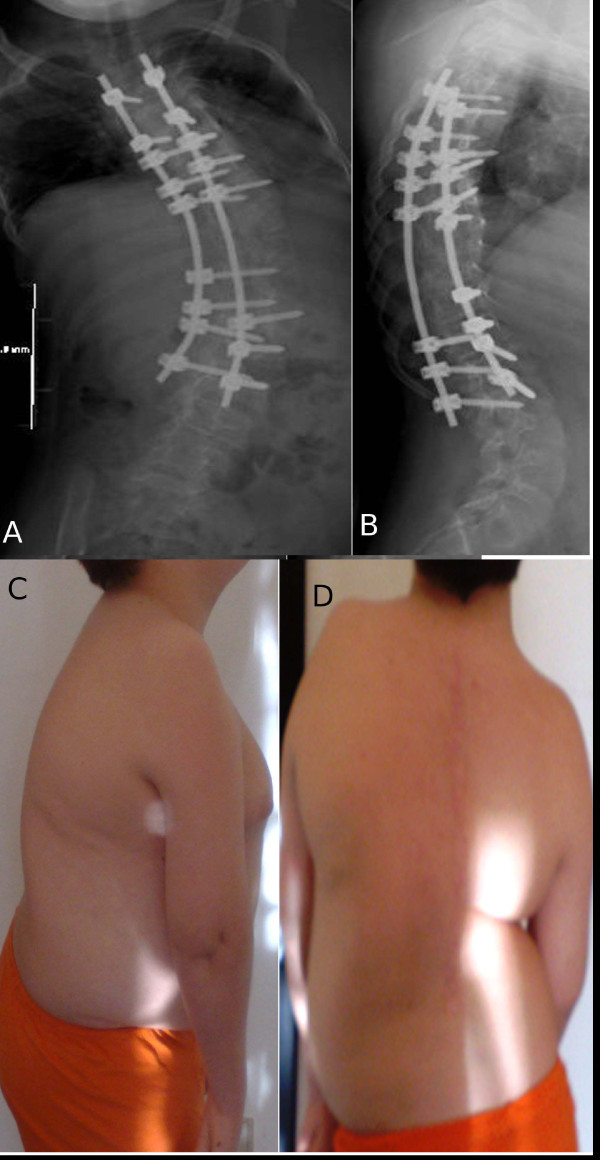
**(A, B, C, D), Case 5: Postoperative radiographs, anteroposterior (A) and laterolateral (B) view and clinical picture at a 2-year follow-up (C and D)**.

**Case 5 **(male, born in 1995, patient with congenital multiple arthrogryposis). In pediatric age he had been operated on twice for congenital clubfoot deformity according to the Codivilla technique and twice for non - traumatic bilateral knee dislocation. At the age of 9, the radiographic evaluation of the spine showed a stiff and structured large thoracolumbar curve of 84° with pelvic obliquity of 5°, while the Risser sign was still 0. Despite the full-time brace treatment since the age of 10, the deformity continued to progress until reaching the following values at the age of 12: thoracolumbar curve with Cobb angle of 100°, pelvic inclination 18°, lumbar lordosis 82° and thoracic kyphosis 73°, with a Risser sign of 1 (Figure 8A, B, C and 8D). The patient was of low stature and overweight with a BMI of 29.5; in addition, a notable restrictive pulmonary deficit was observed and confirmed by the patient's lung capacity (FVC = 54%; FEV1 = 65%). In 2007, posterior instrumented arthrodesis was performed from T4 to L2 using only pedicle screws and achieving a partial correction of the deformity values (thoracolumbar curve 65°, pelvic obliquity 8°, lumbar lordosis 61° and thoracic kyphosis 38°) (Figure 9A and 9B). A Lyonnaise brace was applied for 2 months after surgery. At a 2-year follow-up, no long term complications were observed; radiographs revealed a solid fusion and no sign of mobilization; the young boy maintained his walking capacity in community (Figure 9C and 9D).

**Figure 10:**
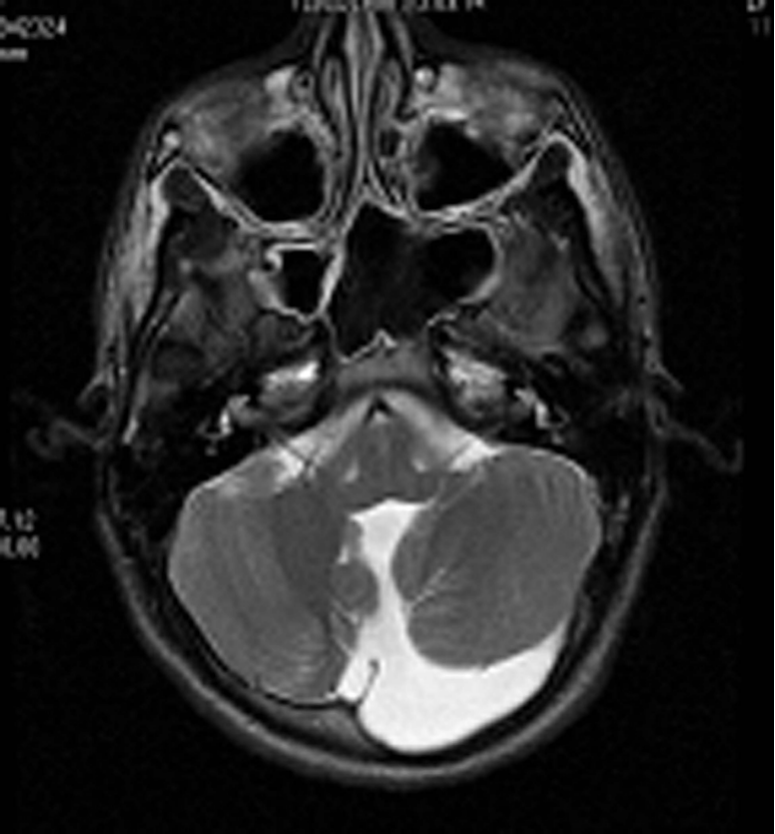
**Case 6: Brain NMRI which highlighted a cystic formation at posterior cerebellar level (Dandy-Walker syndrome)**.

**Case 6 **(male, born in 1990, patient with spinal deformity in congenital multiple arthrogryposis). In pediatric age, he had undergone 5 surgical operations for congenital clubfoot deformity, 2 for bilateral non-traumatic hip dislocation and 1 for palatoschisis. For several years he had also been administered growth hormone. The pulmonary evaluation revealed a restrictive deficit with FVC = 76% and FEV1 = 67%. He was clinically and radiologically evaluated for the first time by the authors' team at the age of 14. No brace had ever been applied and the scoliotic thoracolumbar right convex deformity had reached a Cobb angle of 55° and pelvic obliquity of 3°. By the age of 18, while the Risser sign was 5, the deformity had become stiff and structured with a scoliotic angle equal to 99°, pelvic obliquity 11°, lumbar lordosis 55° and thoracic kyphosis 74°. The subject was also affected with the Dandy-Walker syndrome, as confirmed by the brain NMRI which highlighted a cystic formation at posterior cerebellar level (Figure 10). Before undergoing spine surgery, a dynamic X-Ray evaluation of the cervical spine was performed and showed no sign of cervical instability. Preoperative medullary angiography was also carried out to identify the Adamkiewicz artery. In 2008, at the age of 18, the patient underwent posterior instrumented arthrodesis from T2 to L3 using only pedicle screws. Postoperative values were the following: thoracolumbar scoliotic curve 82°, pelvic obliquity 7°, lumbar lordosis 52° and thoracic kyphosis 60°. No short-term complications were registered. At present, after a 1-year follow-up, the patient's quality of life has improved since he maintains his outdoor walking capacity.

Pre- and postoperative radiographic values are reported in Table 1.

## Results

Five patients underwent one-stage spinal surgery consisting of posterior instrumented arthrodesis. Revision surgery was necessary in 1 patient. Mean operative time was 4 hours (range, 3 to 6), mean intraoperative blood loss was 900 ml (range, 500 to 1500) and mean hospital stay was 10 days (range, 8 to 11). Three minor postoperative complications were encountered, due to intra- and perioperative blood loss as proved by the decrease of the hemoglobin values to below 7.2: they were all successfully treated with adequate blood transfusions. A short-term (3 days after surgery) minor complication was observed in one patient who showed an urticarioid reaction with hyperpiressia, periorbital edema and tachycardia from unidentified cause: prompt and adequate pharmacological antihistaminic therapy led to complete resolution of symptoms. A long-term complication was seen only in one case, C.A., no. 4: the patient had undergone combined single-stage surgery (anterior release and posterior growing rod) at the age of 8 in 1998, he had experienced a distal hook dislodgement after 2 years and had been re-operated on for caudal lengthening of the instrumentation in 2000. At the age of 12, following hook dislodgement, the patient underwent surgery to remove the single bar and have it replaced with the dual rod system; arthrodesis was performed with fresh-frozen banked bone. In the immediate postoperative, pulmonary complications occurred with lung atelectasis, cough with haemoptysis and severe dyspnea: prompt treatment in ICU led to complete resolution of symptoms in 10 days.

Of all the 6 patients, he was the only one who presented a long-term and a major short-term complication. Moreover, the deformity progressed only in his case, on account of the instrumentation removal and subsequent lack of vertebral fusion.

On the whole, 4 cases out of 6 presented no long-term complications, the sixth case having a too short follow-up to be considered, although it is very successful after the first year.

Spine surgery was, however, successful in the other 5 cases, where solid arthrodesis was achieved and no significant curve progression was observed at follow-up. The characteristic rigidity of the deformity did not allow for impressive corrections of the structured curves, except for 2 cases where the postoperative Cobb angle was reduced by 43° and 35°, respectively. Lumbar lordosis and pelvic obliquity were also partially corrected (Table 1). On the whole, these 5 patients reported an improvement in their quality of life after surgery.

## Discussion

Scoliosis in arthrogryposis multiplex congenita is detected within the first year of life, tends to provide resistance to brace treatment and, in most cases, rapidly progresses. This type of spinal deformity often involves pelvic obliquity and lumbar hyperlordosis which, in association with the syndrome-related alterations, bring about a global compromise of the trunk postural alignment and subsequently deteriorate life quality.

Only a few articles in the literature have described the surgical treatment of scoliosis in arthrogryposis multiplex congenita and the number of patients reviewed is also relatively small [9-16]. Scoliosis can be present at birth or develop during early pediatric age. The experience acquired and the literature available lead the present authors to assert that early brace treatment for spinal deformity in arthrogryposis multiplex congenita is often neglected, although its importance is well known. In some cases, however, progression of deformity does not respond to conservative treatment.

Siebold reviewed 5 patients operated on for scoliosis in arthrogryposis and stated that the Milwaukee brace treatment can be useful if started early and followed properly, although results are not always guaranteed. Siebold asserted that spinal fusion is effective in maintaining correction ad preventing curve progression, as further confirmed by the current authors' findings.

Drummond and Mackenzie reported a 28% incidence of scoliosis in a group of 50 patients with arthrogryposis, and described scoliosis and arthrogryphotic deformities involving the lower limbs as disabling. The incidence of congenital scoliosis in their cohort was high, although this finding has not been reported elsewhere: they did not find a single curve pattern to be typical of arthrogryposis, but the majority of patients had either congenital scoliosis or the long "C" curve typical of neuromuscular scoliosis. In the present series no congenital vertebral anomalies were encountered and the most frequent curve pattern was the "C" neuromuscular type, involving most of the thoracic and lumbar column, and observed in 5 out of 6 cases. One patient had a left convex thoracic curve also affecting the first 2 lumbar segments and a low lumbar right convex curve with a less severe rotation.

Herron reviewed 18 patients with scoliosis out of a group of 88 subjects with arthrogryposis. Most of the curves were progressive and became stiff and structured at an early age; progression of the pelvic obliquity coincided with progression of the curve; conservative treatment was ineffective, as curve progression was seen even in children who had been treated with a Milwaukee brace for a mean of 7 years; mean curve progression rate was 5 degrees per year, a rate very similar to that observed during the untreated period of these patients. In the authors' experience, hip surgery did not seem to influence deterioration in pelvic obliquity and scoliosis, and curve correction rates after spinal surgery were disappointing due to the rigidity of the deformity, as confirmed by the findings of the present paper. Herron also suggested prompt and early aggressive spinal surgery when detecting progressive scoliosis in arthrogryposis and advised against temporizing, conservative measures.

Finally, in his series of 46 patients with arthrogryposis Yingsakmongkol found a 65.9% incidence of scoliosis, which is much higher than the data previously reported.

In the present series, the radiographic spinal pictures of early age were available only in 3 out of 6 patients and revealed the presence of the scoliotic deformity associated with arthrogryposis since the first months of life, before the age of 1, as highlighted in the literature. During pediatric age, the Cobb angle and the other deformity indexes often progress into a stiff and extensive deformity. The main reason for changing the technique of surgery was the time period of 21 years, the case series refers to.

Brace treatment remains controversial as spine deformities in arthrogryposis tend to resist brace treatment and progress rapidly in most cases. Nonetheless, the present authors firmly believe that full-time brace treatment should always be attempted since early age and in all patients, since there are no verified criteria to predict curve progression and response to conservative treatment.

The authors' experience and the scientific evidence of the aggressive evolution of this deformity suggest that in case of brace treatment failure, prompt surgery should be considered, to avoid further perpetuation of the deformity with subsequent remarkable functional disability and poor health.

Spinal instrumented arthrodesis has proved to be capable of delaying progression of the deformity with satisfactory results over time. The evolution of surgical procedures, modern spinal instrumentation with pedicle screws and advanced anesthesia techniques enable significant corrections of spinal deformities to be achieved. New spinal implants and lengthening techniques without arthrodesis should be taken into consideration when deciding to perform surgery at an early age, in order to prevent the deformity from becoming irreversibly stiff.

## Competing interests

The authors declare that they have no competing interests.

## Authors' contributions

TG performed surgeries, conceived the study and coordinated the work; KM drafted the manuscript and participated in its design and coordination; EP analyzed data; FL collected and reviewed clinical and radiographic charts; FV collected and reviewed clinical and radiographic charts; EM assembled the references; SC performed surgeries; MDS performed surgeries. All authors read and approved the final manuscript.

## Consent section

Written informed consent was obtained from the patient for publication of this case report and accompanying images. A copy of the written consent is available for review by the Editor-in-Chief of this journal.
